# Selective peroxisome proliferator-activated receptorα modulators (SPPARMα): The next generation of peroxisome proliferator-activated receptor α-agonists

**DOI:** 10.1186/1475-2840-12-82

**Published:** 2013-05-31

**Authors:** Jean-Charles Fruchart

**Affiliations:** 1 Fondation coeur et arteres, 96, rue Nationale, 59000, Lille, France

**Keywords:** Peroxisome proliferator-activated receptor, Fibrate, SPPARM, K-877, Cardiovascular, Diabetes, Metabolic syndrome

## Abstract

Dyslipidemia is a major risk factor for cardiovascular (CV) disease – the primary cause of death, worldwide. Although reducing levels of low-density lipoprotein-cholesterol can significantly reduce CV risk, a high level of residual risk persists, especially in people with obesity-related conditions, such as metabolic syndrome and type 2 diabetes mellitus. Peroxisome proliferator-activated receptor alpha- (PPARα-) agonists (e.g. fibrates), play a central role in the reduction of macro- and microvascular risk in these patients. However, the currently available fibrates are weak (PPARα-agonists) with limited efficacy due to dose-related adverse effects. To address this problem, a new generation of highly potent and selective PPARα-modulators (SPPARMα) is being developed that separate the benefits of the PPARα-agonists from their unwanted side effects. Among these, aleglitazar (a dual PPARα/γ agonist) and GFT505 (a dual PPAR α/δ agonist) have recently entered late-phase development. Although both compounds are more potent PPARα-activators than fenofibrate *in vitro*, only aleglitezar is more effective in lowering triglycerides and raising high-density lipoprotein-cholesterol (HDL-C) in humans. However, it is also associated with a potential risk of adverse effects. More recently, a highly potent, specific PPARα-agonist (K-877) has emerged with SPPARMα characteristics. Compared to fenofibrate, K-877 has more potent PPARα-activating efficacy *in vitro*, greater effects on triglycerides- and HDL-C levels in humans, and a reduced risk of adverse effects. If successful, K-877 has the potential to supersede the fibrates as the treatment of choice for patients with residual CV risk associated with metabolic syndrome and type 2 diabetes.

## Introduction

Dyslipidemia is a major risk factor for cardiovascular (CV) disease – the leading cause of morbidity and mortality in the developed world [1]. Lowering low-density lipoprotein-cholesterol (LDL-C) levels using lifestyle change and pharmacotherapy can significantly reduce CV risk in people with and without cardiometabolic diseases, such as metabolic syndrome (MetS) and type 2 diabetes mellitus (T2D) [2-4]. However, the risk of macrovascular events in those attaining the maximum levels of LDL-C reduction is only reduced by around 30%, leaving substantial residual risk [2]. Moreover, the risk of microvascular events in people with MetS or T2D can only be reduced by approximately 50% using the current standard of care (intensive treatment to reduce LDL-C, blood pressure and blood glucose) [5]. Recent studies demonstrate that low levels of high-density lipoprotein-cholesterol (HDL-C) (<1.0 mmol/L; 40 mg/dL) and high levels of triglycerides (TGs) (≥1.7 mmol/L; 150 mg/dL) are independent risk factors for both macro- and microvascular diseases [6-8]. Consequently, treatment guidelines highlight the importance of targeting these risk factors in addition to LDL-C [9]. This is particularly important for patients with cardiometabolic diseases who often have atherogenic dyslipidemia – a triad of lipid abnormalities that includes low levels of HDL-C, high levels of TG and a preponderance of atherogenic small, dense LDL particles [9-11]. In addition, patients with these lifestyle-related conditions typically present with chronic inflammation and other obesity-related risk factors, such as insulin resistance and hyperglycemia. The ideal strategy for reducing CV risk in patients with MetS or T2D should therefore encompass many aspects of cardiometabolic control in addition to lipid homeostasis [9,11].

### Peroxisome proliferator-activated receptor alpha (PPARα) agonists

#### Effects in vitro and in vivo

The PPARα-agonists (e.g. fibrates) play a central role in reducing plasma concentrations of TG-rich lipoproteins, increasing HDL-C levels and reducing vascular inflammation in people with atherogenic dyslipidemia [10,12,13]. Furthermore, PPARα-agonists can increase the stability of atherosclerotic plaques, reduce the risk of atherothrombosis, slow the progression of intimal hyperplasia following surgery, and reduce hepatic fat accumulation leading to non-alcoholic steatohepatitis/fatty liver disease (NASH/NAFLD) [14]. The net effect of these actions is a significant reduction in macrovascular events in people with, but not without, atherogenic dyslipidemia [12,13,15-18], and significant reductions in T2D-related microvascular events [19-22]. For example, the Fenofibrate Intervention and Event Lowering in Diabetes (FIELD) study demonstrated a 27% reduction in the risk of major CV events in patients with atherogenic dyslipidemia (P<0.005) [23], a 14% reduction in albuminuria progression over 5 years (P<0.001) [21], a 36% reduction in the risk of first amputation (P=0.02) [19], and a 31% reduction in the need for first laser treatment (P=0.0002) [20]. Although fibrates are the only lipid-lowering drugs to demonstrate clinically significant benefits for both macro- and microvascular disease in people with T2D, they are weak PPARα-agonists whose efficacy is partly limited by dose-dependent side effects. Commonly-reported adverse events (AEs) include elevations in markers for CV disease (e.g. homocysteine), renal disease (e.g. creatinine) and liver dysfunction (e.g. alanine aminotransferase [ALT] and γ-glutamyl transpeptidase) [12,13,24]. There is therefore an unmet medical need for a new generation of more potent PPARα-agonists with a lower potential for AEs.

#### Mechanism of action

Three PPAR isoforms have been identified (PPARα, γ and δ), each encoded by a separate gene [25]. PPARα is abundant in highly active metabolic tissues including the liver, kidney, heart, muscle, brown adipose and macrophages, whereas PPARγ is predominantly found in adipose tissue, macrophages and large intestine. In contrast, PPARδ (also called PPARβ) is ubiquitously expressed.

PPARs are activated when endogenous ligands (e.g. prostaglandins, leukotrienes, free fatty acids) or synthetic PPAR agonists (e.g. glitazones, fibrates) bind to the lipid-binding domain enabling heterodimerisation with a ligand-activated retinoid X-receptor (RXR) [26-30]. This process triggers a conformational change, leading to the transrepression or transactivation of target genes. During transrepression, the activated PPAR binds to cytokine-activated transcription factors, such as nuclear factor kappa B or activator protein-1 [31,32]. Under normal conditions, these transcription factors induce the synthesis of proteins involved in the inflammatory response. PPARs can inhibit this process by blocking the interaction between activated transcription factors and the promoter region of the target gene, thereby preventing transcription and reducing inflammation. During transactivation, the activated PPAR binds to a specific sequence of nucleotides (the PPAR receptor response element) upstream of the target gene. A cofactor (either a coactivator or a corepressor) renders the PPAR complex ‘transcriptionally active’ and gene transcription begins. A large number of genes carry response elements for PPARs. Targets for PPARα include key genes involved in lipid metabolism, such as *apo A-I, A-II* and *A-V, LPL* and *SR-BI*, whereas PPARγ targets include genes involved in obesity and insulin resistance, such as *ADIPOQ* and *ADRP.* Consequently, PPARα primarily regulates lipid homeostasis [14], whereas PPARγ largely regulates adipogenesis and glucose homeostasis [33]. However, the precise receptor-mediated response depends on the individual agonist and the tissue in which it is expressed.

**Figure 1:**
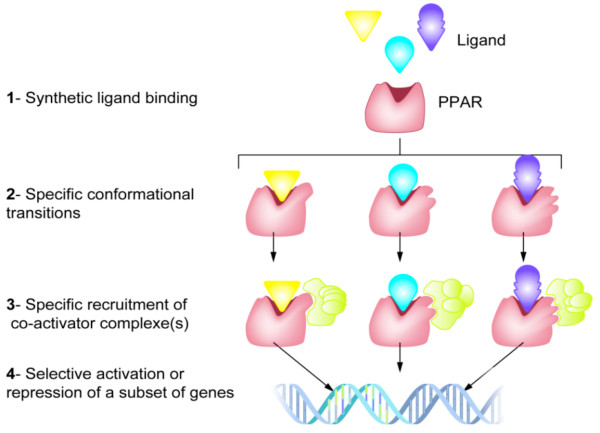
**PPAR-agonists have the potential to trigger different biological responses via the same receptor**[34]**.**

**Figure 2:**
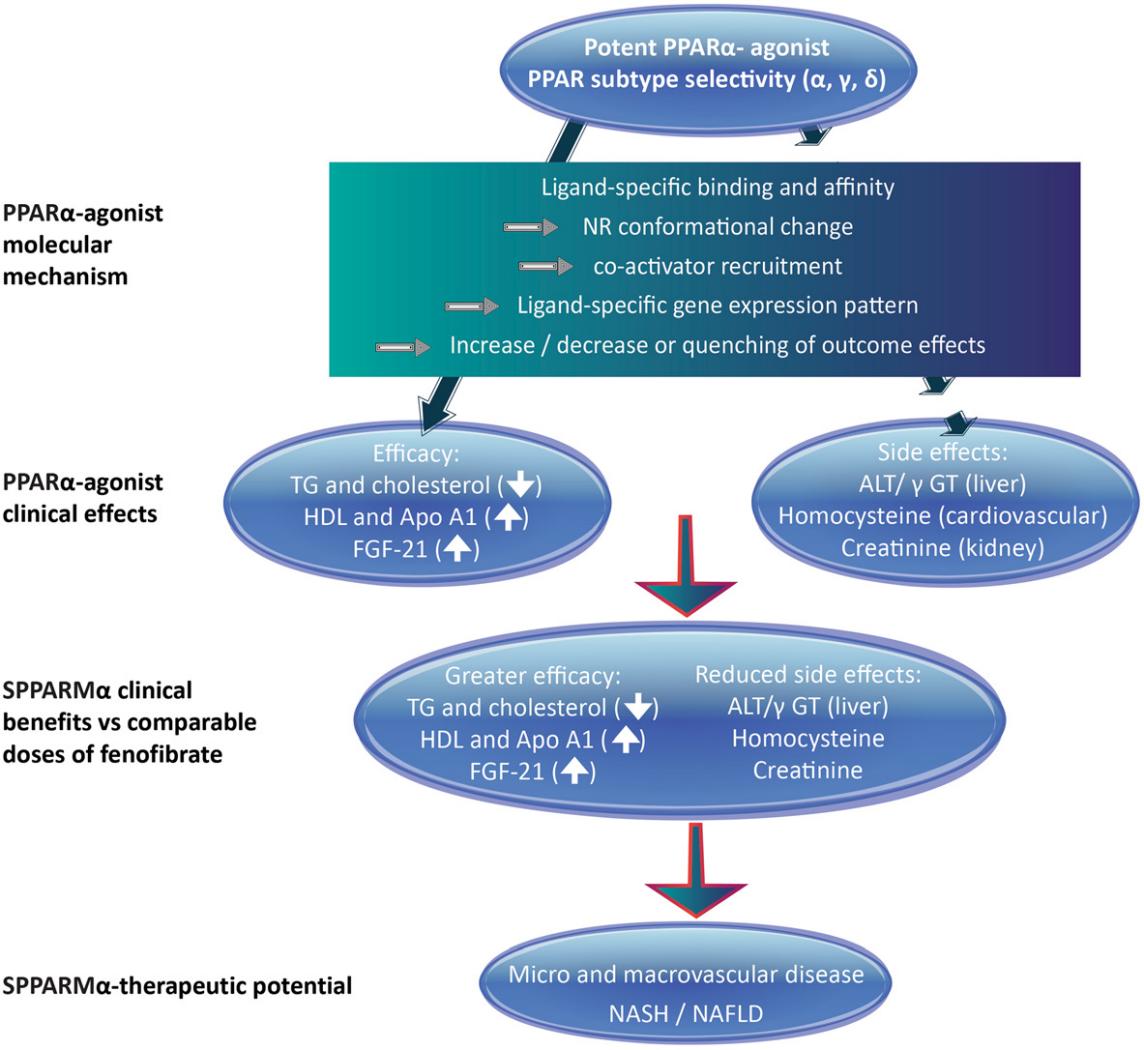
**SPPARMα characteristics.** ALT alanine aminotransferase; Apo apolipoprotein; FGF-21 fibroblast growth factor 21; γGT γ-glutamyl transpeptidase; HDL high-density lipoprotein; NASH/NASFL non-alcoholic steatohepatitis/fatty liver disease; NR nuclear receptor; TG triglyceride.

Compared to other nuclear receptors, PPARs have a large lipid-binding pocket capable of encompassing a range of endogenous ligands [34]. This provides a variety of potential contact points that, when occupied, can trigger different conformational changes. Since each PPAR conformation is associated with a unique cofactor recruitment pattern, the same PPAR subtype has the potential to induce a wide range of biological effects depending on its agonist (Figure 1). The number of possible effects is further increased by the fact that PPAR-activation requires both a specific ligand and an activated RXR complex, the concentration and affinity of which can vary between tissues, organs, and individuals. It is therefore possible for a ligand to act as a full agonist in a tissue where sufficient concentrations of a specific coactivator are present, and as a partial agonist in another tissue with higher concentrations of the same coactivator. For example, gemfibrozil and fenofibrate (both PPARα-agonists) have a similar impact on levels of HDL-C, TG and small dense LDL but, whereas fenofibrate (a full PPARα-agonist) has additional benefits on apolipoprotein A-I (apo A-I) and fibrinogen levels [35], gemfibrozil (a partial PPARα-agonist) has little or no effect [36]. Moreover, whereas most fibrates specifically activate PPARα, bezafibrate is a pan PPAR-agonist, activating all three PPAR subtypes (α, γ and δ) at comparable doses [37]. Although this effect can be exploited to increase the number of biological benefits, it can also be associated with an increased risk of unwanted side effects. Consequently, the development of ideal PPAR-agonists involves a series of *in vitro* and *in vivo* assays to identify the most potent molecules that differentially induce receptor-mediated beneficial effects in specific tissues whilst avoiding unwanted side effects [34]. This is the pharmacological basis for the selective PPARα modulators (SPPARMs) (Figure 2).

### The next generation of selective PPARα modulators

The concept of SPPARMs (and other selective nuclear receptor modulators) was initially based on the paradigm of tamoxifen, a pioneering selective estrogen receptor modulator that exhibits anti-estrogenic activity in the mammary gland and partial pro-estrogenic activity in bone and uterus [38]. The observed increase in the incidence of uterine cancer with prolonged tamoxifen use led to the development of raloxifene, a second-generation estrogen receptor modulator with highly selective, tissue-specific activity that avoids uterotrophic effects. Since then, selective modulators have been identified for most classes of ligand-modulated nuclear receptor [39]. For example, a number of PPARγ-agonists with SPPARM properties (e.g. INT131 and MK0533) have been developed for the treatment of T2D [40,41]. Preclinical studies show that these molecules have comparable or more potent antidiabetic benefits to the gold-standard treatment, pioglitazone, with fewer AEs [41]. More recently, the SPPARM concept has been extended to other PPAR subtypes, including PPARα. If successful, these molecules have the potential to become superior therapeutics for the treatment of CV risk associated with MetS and T2D.

### Dual PPAR-agonists with SPPARMα properties

Whereas PPARα-agonists can improve lipid control and PPARγ-agonists can improve glucose homeostasis, dual PPARα/γ-or α/δ agonists can potentially be used to treat a range of cardiometabolic imbalances at the same time. Moreover, these molecules can be designed to offset each other’s side effects [26,42]. For example, weight increases due to the adipogenic effects of PPARγ-agonists can potentially be negated by PPARα-mediated increases in lipid catabolism. Although many dual PPAR-agonists have undergone clinical trials, none have progressed past Phase III due to unresolved safety concerns [42]. Muraglitazar, tesaglitazar, ragaglitazar, TAK559 and KRP292, for example, were discontinued due to an increased risk of CV events, renal dysfunction, weight gain and edema. However, two dual agonists with possible SPPARM properties (aleglitazar and GFT505) have recently entered late-phase development.

**Figure 3:**
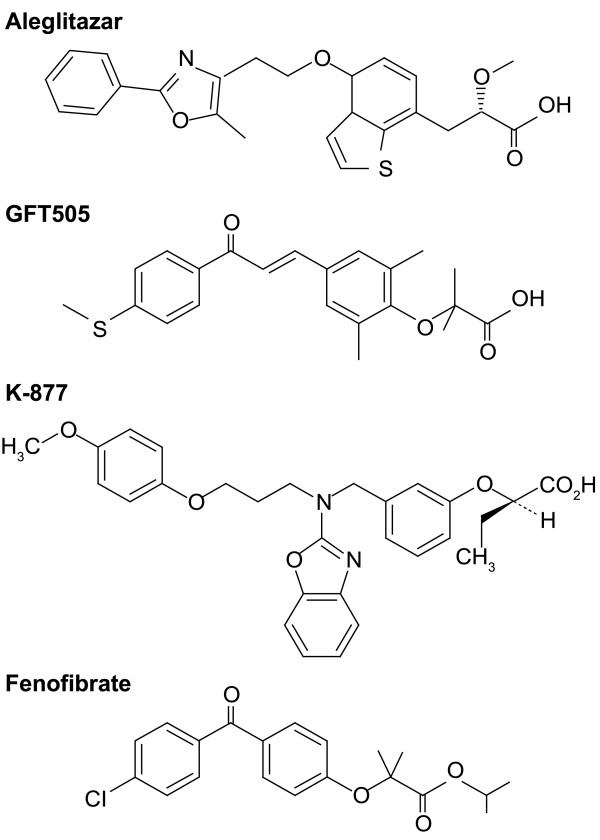
Chemical structures of PPARα agonists.

Aleglitazar (Figure 3) is a dual PPARα/γ-agonist developed by Roche Holding for the treatment of residual CV risk in people with T2D [26,43,44]. Compared to pioglitazone, it is a more potent PPARγ-agonist with greater effects on glucose homeostasis and a reduced risk of AEs [26]. In addition to its PPARγ agonist properties, aleglitazar is a more potent PPARα-agonist than fenofibrate, both *in vitro* (Table 1) [26] and *in vivo* (Table 2) [44]. For example, a Phase II study in 332 people with T2D showed that 16 weeks treatment with aleglitazar 150 μg once daily (QD) was associated with significant placebo-adjusted changes in both TG (−43.4%) and HDL-C (+20.7%) (Table 2) [44]. In comparison, 16 weeks treatment with fenofibrate 200mg QD in the FIELD study reduced TG levels by 28.6% vs. placebo and increased HDL-C by 5.1% (Table 2) [12]. Although aleglitazar has greater PPARα-mediated effects on the lipid profile than fenofibrate, it is associated with several potential safety concerns, including weight gain, peripheral edema and increased creatinine kinase levels with corresponding decreases in estimated glomerular filtration rate [43-46]. Since it is unable to completely separate the beneficial effects of the PPARα-agonists from their unwanted AEs, aleglitazar is unlikely to be classified as a true SPPARMα.

**Table 1:** Effects of PPARα-agonists on PPAR transcriptional activity

**Compound**		**Aleglitazar**[26]	**GFT505**[48]	**K-877**[52]	**Fenofibrate**[26,52]
PPARα	EC_50_ (nM)	5	10 to 20	1	14,000 [52]; 22,400 [26]
PPARγ	EC_50_ (nM)	9	NA	2,300	~100,000
PPARδ	EC_50_ (nM)	376	100 to 150	1,000	not activated

EC50 effective concentration inducing 50% response; NA not available.

**Table 2:** Effects of PPARα-agonists on triglycerides (TG) and high-density lipoprotein-cholesterol (HDL-C) levels

**Compound**	**Aleglitazar**	**GFT505**		**K-877**	**Fenofibrate**	
**Study**	**SYNCHRONY**[43,44]	**S1**[48]	**S2**[48]	**K-877 P2**[50]	**FIELD**[12]	**K-877**[50]
**Dosage**	**150 μg QD**	**80 mg QD**	**80 mg QD**	**100 μg BID**	**200 mg QD**	**100 mg QD**
**Time point (weeks)**	**16**	**4**	**4**	**12**	**16**	**12**
TG						
Baseline (mg/dL)	157.7*	283.5	194.9	290.9	172.8	325.2
Change vs. baseline	NA	−51.4	−62.9	NA	NA	NA
(mg/dL)						
% change vs. baseline	−29.7**	NA	NA	−41.4	NA	−30.7
% change vs. placebo	−43.4**	−16.7**	−24.8**	−69.9	−28.6	−59.2
HDL-C						
Baseline (mg/dL)	46.8	34.8	46.4	40.9	42.5	40.1
Change vs. baseline	NA	2.7	3.1	NA	NA	NA
(mg/dL)						
% change vs. baseline	25.1**	NA	NA	16.9	NA	13.0
% change vs. placebo	20.7**	7.8**	9.3**	18.2	5.1	14.3

BID twice daily; NA, not available; QD once daily;

Where necessary, values were multiplied by 88.6 for TG and by 38.67 for HDL-C to convert from mmol/L to mg/dL.

Values are expressed as means unless otherwise stated.

*Median.

**Least squares means.

Another dual agonist with SPPARM characteristics is GFT505 (Figure 3) [47,48]. Developed by GenFit, GFT505 is a dual PPARα/δ-agonist with preferential activity on PPARα (Table 1) [48]. Phase II studies in people with combined dyslipidemia and abdominal obesity (N = 97) or prediabetes (N = 47) showed that 28 days treatment with GFT505 80 mg QD was associated with significant reductions in ALT and γ-glutamyl transpeptidase levels (Table 3) [48], suggesting potential benefits for GFT505 in patients with NASH/NAFLD. This is particularly important because, although the obesity-related incidence of fatty liver disease continues to rise, no effective treatments are currently available [49]. However, although GFT505 was associated with significant placebo-adjusted changes in the levels of TGs (−16.7% to −24.8%) and HDL-C (+7.8% to +9.3%) [48], the magnitude of the effects was similar to those obtained in clinical studies with fenofibrate (Table 2) [12]. GFT505 is, therefore, unlikely to be classified as a true SPPARMα due to its lack of superior lipid-modifying efficacy vs. fenofibrate.

**Table 3:** Safety parameters

**Compound**	**Aleglitazar**	**GFT505**		**K-877**	**Fenofibrate**	
**Study**	**SYNCHRONY**[43,44]	**S1**[48]	**S2**[48]	**K-877 P2**[50]	**FIELD**[12]	**K-877**[50]
**Dosage**	**150 μg QD**	**80 mg QD**	**80 mg QD**	**100 μg BID**	**200 mg QD**	**100 mg QD**
**Time point (weeks)**	**16**	**4**	**4**	**12**	**16**	**12**
ALT (UI/L)	NA	−7.1	−2.1	−7.6	NA	−4.2
γGT (UI/L)	NA	−11.0	−6.0	−24.6	NA	0.0
Serum creatinine (mg/dL)	NA	0.038*	0.057*	0.013	NA	0.086
Homocysteine (nmol/mL)	NA	1.71	−0.8	0.16	NA	2.21

ALT alanine aminotransferase; BID twice-daily; γGT γ-glutamyl transpeptidase; NA not available; QD once daily.

Data are expressed as mean changes from baseline.

*To convert from μmol/L to mg/dL, values were divided by 88.4.

### K-877: The first of the SPPARMαs

K-877 (Kowa Co. Ltd.) is a highly potent and selective PPARα-agonist with SPPARM properties (Figure 2) [50-52]. It is currently undergoing Phase I trials in Europe and the USA and Phase III trials in Japan for the treatment of atherogenic dyslipidemia. K-877 contains an acidic region similar to that found in other PPARα-agonists but, to enhance PPARα activity and selectivity, unique benzoxazole and phenoxyalkyl side-chains have been added (Figure 3) [53]. Cell-based transactivation assays using hPPAR-GAL-4 chimeric receptors confirmed that, compared to fenofibrate, K-877 is a more potent PPARα-agonist, with a high degree of PPAR subtype-selectivity (Table 1) [52].

Pre-clinical studies in animal models for obesity demonstrated that low doses of K-877 (0.3-3.0 mg/kg) had a greater TG-lowering efficacy than 1,000-fold higher doses (300 mg/kg) of fenofibrate, an effect that was accompanied by higher levels of plasma FGF-21 [52]. Furthermore, K-877 0.01-0.1 mg/kg significantly reduced atherosclerotic lesion area in LDL receptor-null mice fed a Western diet, and significantly reduced the expression of *TNF-α* and *MCP-1* genes. Although there were slight reductions in all three of these parameters with fenofibrate 100 mg/kg, the benefit was not significant.

More recently, a Phase II 12-week dose-finding study (N = 224) showed that K-877 100 μg twice daily (BID) was well tolerated and had a greater lipid modifying efficacy than fenofibrate 100mg QD in patients with atherogenic dyslipidemia (13% with T2D) [50]. The incidences of AEs (47.4% with K-877, 47.2% with placebo and 56.8% with fenofibrate) and adverse drug reactions (5.3% with K-877, 8.3% with placebo and 10.8% with fenofibrate) were similar for K-877 and placebo and slightly higher for fenofibrate. No serious AEs were reported for K-877. As expected, fenofibrate was associated with significant increases vs. baseline in serum creatinine and homocysteine levels and little or no effect on ALT or γ-glutamyl transpeptidase levels (Table 3). In contrast, K-877 100 μg BID had little or no effect on CV or renal markers, and hepatic markers were improved. Together, these results suggest that K-877 has a better safety and tolerability profile than fenofibrate and might be useful for people with NASH/NAFLD.

As suggested by pre-clinical studies, the Phase II study demonstrated greater changes from baseline in fasting plasma TG and HDL-C levels with K-877 100 μg BID than with fenofibrate in people with atherogenic dyslipidemia (Table 2). Compared to placebo, significant changes in TG, HDL-C, non-HDL-C and very low-density lipoprotein were observed for K-877. Although not directly compared, all changes were more pronounced with K-877 than with fenofibrate. In addition, K-877 was associated with greater beneficial changes in the size of atherogenic lipoproteins than fenofibrate [50], suggesting that K-877 has the potential to improve lipoprotein quality as well as quantity.

Results from a ‘Cookie test’ [54] subanalysis (N = 143) showed that 12 weeks treatment with K-877 had a significantly greater potential than fenofibrate to improve postprandial hyperlipidemia, a major risk factor for CV disease in people with MetS or T2D [51]. For example, K-877 suppressed the postprandial increase in TG, apo B-48 and remnant-like particle cholesterol, a major risk factor for ischemic heart disease [55]. Although no clear between-group differences in glucose or insulin levels were observed in the substudy, results from the Phase II trial showed that K-877 was associated with significant increases in plasma levels of FGF-21. Similarly, K-877 was associated with increased levels of *FGF21* gene expression in the livers of LDL receptor-null mice and increased levels of plasma FGF-21 in ZF rats [52]. These observations are important because *FGF21* expression in white adipose tissue increases in response to feeding and is generally associated with weight loss, antidiabetic and hypolipidemic effects in animal models of T2D and obesity [56,57]. Further studies are required to fully understand the implications of these results and to examine the effects of K-877 on vascular inflammation in humans.

## Conclusions

Fibrates play a central role in the reduction of macro- and microvascular risk associated with MetS and T2D [12,13,20-23]. However, they are weak PPARα agonists with limited efficacy due to dose-related AEs. To address this problem, a new generation of tissue-specific PPARα agonists — the SPPARMαs — is being developed that separates the receptor-mediated beneficial effects of the PPARα agonists from their unwanted side effects. Although a number of dual PPARα/γ and α/δ-agonists have been developed with SPPARMα characteristics, most are associated with unresolved safety issues [26,42-44] or fail to provide a superior efficacy vs. standard treatment [47,48].

Recently, a highly specific PPARα-agonist (K-877) has emerged with SPPARM properties. Although K-877 has not been compared with fenofibrate in head-to-head studies, it is approximately 10,000-fold more potent than fenofibrate *in vitro*[52], has a greater lipid-modifying efficacy at considerably lower doses both in animal models for obesity/T2D and in humans, and is associated with an improved safety/tolerability profile [50-52]. In addition, K-877 100 μg BID is associated with beneficial changes in markers for liver disease. This suggests that, in addition to improving lipid parameters in people with cardiometabolic diseases, K-877 might also be useful for the prevention of NASH/NAFLD. Further studies are required to characterize the effects of K-877 on the recruitment of cofactors and gene expression to clarify the precise molecular mechanism, and to investigate the long-term safety and clinical efficacy of K-877 in people with atherogenic dyslipidemia. However, results to date suggest that K-877 is the first true member of the SPPARMα family. If successful, this next generation of PPARα-agonists has the potential to supersede fibrates as the treatment of choice in patients with atherogenic dyslipidemia and could have a major impact on the management of residual macro- and microvascular risk associated with MetS and T2D.

## Abbreviations

AE: Adverse event; ALT: Alanine aminotransferase; Apo: Apolipoprotein; BID: Twice daily; CV: Cardiovascular; FIELD: Fenofibrate intervention and event lowering in diabetes; HDL-C: High-density lipoprotein-cholesterol; LDL-C: Low-density lipoprotein-cholesterol; MetS: Metabolic syndrome; NAFLD: Non-alcoholic fatty liver disease; NASH: Non-alcoholic steatohepatitis; PPAR: Peroxisome proliferator-activated receptor; QD: Once daily; SPPARM: Selective peroxisome proliferator-activated modulator; T2D: Type 2 diabetes; TG: Triglyceride.

## Competing interests

J-C Fruchart has received honoraria as a consultant for SMB laboratories, McCain and Kowa Co. Ltd.

## Authors’ contribution

J-C Fruchart confirms he is the sole author of this review.

## Authors’ information

JCF is Past-President of the International Atherosclerosis Society. He is currently President of the Residual Risk Reduction Initiative (http://www.r3i.org/pg/1/), an international, academic, multidisciplinary non-profit organization, which is focused on addressing the high residual risk of macro- and microvascular complications in patients with atherogenic dyslipidemia.
